# Supplementary material for: Banana 0.9: An open-source, reproducible medical imaging system for low-resource gastric cancer screening
Source: PLoS One. 2026 Feb 24;21(2):e0339892. doi: 10.1371/journal.pone.0339892 (PMC12931783; doi:10.1371/journal.pone.0339892)
Supplement: Attachment — Submitted filename: Review report (v1.1).docx [file pone.0339892.s002.docx]

Thank you for submitting this work to PLOS ONE. Your manuscript addresses an important global health need affordable, reproducible medical imaging workflows for gastric cancer screening in low-resource settings. The open-source orientation is valuable and timely. The comments below are intended to help you strengthen methodological rigor, transparency, and empirical validation so that the paper can better fulfill its stated goals and meet the journal’s standards.

**Major Comments**

1. **Clinical/External Validation is Missing**

The study relies on simulations and TCGA-STAD without validation on independent, real-world clinical datasets. Please include external validation (e.g., multicenter hospital data or recognized open clinical repositories) to establish generalizability.

2. **Performance Claims Appear Overstated Without Benchmarks**

Reported improvements (65–85%) require benchmarking against state-of-the-art methods (e.g., U-Net variants, transformer-based models) using standard metrics (Dice, AUC, sensitivity/specificity, PPV/NPV). Claims should be tempered unless supported by comparative results.

3. **Statistical Design and Uncertainty Quantification**

Monte Carlo simulations are mentioned but statistical methods are under-specified. Please report variance, CIs, sample size justifications, error analysis, and reproducibility statistics. Clarify random seeds, number of runs, and any stratification.

4. **Algorithmic Transparency and Reproducibility Materials**

The HU-based segmentation and Bayesian updating are not described at a level enabling replication. Provide pseudocode, parameter ranges, thresholds, and configuration files or scripts (e.g., Docker/Conda) to ensure end-to-end reproducibility.

5. **Positioning Within Prior Work**

The literature review under-represents reproducible pipelines, federated learning, and low-power/edge AI for medical imaging. Expand the review to critically situate the contribution and clarify how Banana 0.9 advances the state of the art.

6. **Ethics, Licensing, and Data Governance**

Even for secondary/synthetic data, specify dataset identifiers, license terms, and governance (IRB/ethics determinations as applicable). Clarify the practical implications of the CC BY-NC 4.0 license for clinical research and deployment.

7. **Reproducibility Evidence**

Since reproducibility is central, add a reproducibility assessment (e.g., independent replication across machines/OS, cross-platform containers, or third-party validation) and report concordance metrics.

8. **Limitations Need Stronger Treatment**

Expand on biases from synthetic or low-resolution data, limited diversity, and hardware simplifications. Discuss how these factors may affect sensitivity/specificity and clinical applicability.

9. **Tables/Figures: Captions, Provenance, and Flow**

Add complete captions (units, data sources, methods), ensure clear cross-referencing in text, and include a pipeline flow diagram with module I/O, versioning, and checkpoints for QA.

10. **Roadmap for Future Releases**

References to “Banana 1.0” should be accompanied by a concrete roadmap (validation milestones, community governance, testing protocols, and plans for clinical collaboration).

**Minor Comments**

- **Title:** Consider a research-focused phrasing rather than a versioned software label (“0.9”), unless this is explicitly a software descriptor paper.
- **Abstract:** Specify dataset(s), quantitative metrics, and limitations; structure as Background, Methods, Results, Conclusions.
- **Keywords:** Consider adding “CT segmentation,” “Monte Carlo,” and “AI reproducibility.”
- **Introduction:** Reduce repetition of “low-resource settings”; expand comparative discussion with prior open frameworks.
- **Methods:** Include algorithmic flow diagrams; ensure figure numbering consistency.
- **Results:** Provide uncertainty (SD or CI) for all metrics; ensure each table/figure is referenced in the narrative.
- **Discussion:** Temper claims and add comparative error analysis.
- **References:** Reduce self-citations where possible; add targeted works on open, reproducible medical AI.
- **Figures/Tables:** Include resolution, dataset versioning, and software/library versions.
- **License Clarification:** Explain clinical reuse boundaries under CC BY-NC 4.0.

**Recommendation:**

**Major Revision / Resubmission for Review.**

In summary, while the manuscript addresses a high-value problem with a commendable open-source philosophy, it currently falls short in three critical areas: (i) external/clinical validation, (ii) comparative benchmarking with robust uncertainty reporting, and (iii) detailed, reproducible algorithmic documentation. Addressing these gaps alongside the strengthened literature positioning, ethics, data-governance clarifications, enhanced figure/table documentation, and a concrete roadmap would substantially improve the manuscript’s scientific credibility and practical relevance for PLOS ONE.
