# Supplementary material for: Banana 0.9: An open-source, reproducible medical imaging system for low-resource gastric cancer screening
Source: PLoS One. 2026 Feb 24;21(2):e0339892. doi: 10.1371/journal.pone.0339892 (PMC12931783; doi:10.1371/journal.pone.0339892)
Supplement: Attachment — Submitted filename: Response_to_Reviewers_PONE-D-25-49901.docx [file pone.0339892.s003.docx]

# ****Response to Reviewers****

**Manuscript ID:** PONE-D-25-49901
**Title:** Banana 0.9: An Open-Source, Reproducible Medical Imaging System for Low-Resource Gastric Cancer Screening

## ****Dear Academic Editor and Reviewers,****

We sincerely thank the Academic Editor and both reviewers for their detailed, constructive, and insightful comments. Their feedback significantly improved the scientific rigor, structure, and reproducibility of the revised manuscript.

We have carefully addressed every point raised, and all changes are incorporated into the revised manuscript. Below we provide a **point-by-point response**, indicating precisely where modifications were made.

Line numbers refer to the **clean revised manuscript** unless specified otherwise.

# ****Response to Academic Editor Requirements****

### ****1. PLOS ONE style & formatting****

All formatting issues have been corrected:

Updated file naming

Added track-changes version

Revised affiliations

Relocated tables into the main text

Removed figures from the manuscript body (Figure 1 uploaded separately)

**2. Code-sharing compliance**

All author-generated code is:

Publicly available on GitHub

Released under CC BY-NC 4.0

Includes scripts, configs, and logs

Contains a reproducible CPU-only pipeline

Added explicit statements in **Data & Code Availability** (Lines 658–662).

**3. Tables merged into main manuscript**

Tables 1 & 2 now appear in the Results section (Lines 320–337).
All standalone table files have been removed.

**4. Data availability clarification**

We explicitly confirm:

Full data for TCGA-STAD is publicly available

External dataset (AbdomenCT-1K) is publicly available

All logs, preprocessing outputs, and hashes are provided

No private/personal data were used
Updated Data Availability section (Lines 658–662).

### ****5. Affiliation corrections****

All authors are now assigned correct institutional affiliations.
(Current affiliation labels added where needed.)

### ****6. Removal of inline figures****

All figures have been removed from the manuscript file.
Figure files uploaded individually as TIFF.

### ****7. Missing Tables 1 & 2****

Tables 1 and 2 are now included and cross-referenced correctly (Lines 320–337).

### ****8. Optional citations from reviewers****

Relevant literature was added where appropriate (Lines 96–115 and throughout).

# ****Response to Reviewer #1****

We sincerely appreciate Reviewer #1’s positive evaluation and constructive suggestions.

### ****Reviewer #1 Comment 1 – Need for empirical validation****“Explain how Banana 0.9 will be validated using larger or future clinical datasets.”

**Response:**
We have expanded the **Future Work** section (Lines 624–642) to include a clear roadmap:

Planned multi-center collaborations

Integration with real biomarker datasets

Prospective validation cohorts

External testing on additional cancer types

**Reviewer #1 Comment 2 – Dataset Constraints / TCGA Limitations**“Discuss more explicitly how TCGA preprocessing affects generalizability.”

**Response:**
We added a strengthened paragraph in **Limitations** (Lines 574–604) describing:

TCGA preprocessing variability

Scanner heterogeneity

Limited demographic diversity

Potential HU-range inconsistencies

### ****Reviewer #1 Comment 3 – Quantitative comparison with nnU-Net / DeepMedic****

“Comparisons were brief; add more quantitative benchmarks.”

**Response:**
Expanded comparisons in **Comparison to Existing Tools** (Lines 398–429), including:

Dice = 0.83 (95% CI 0.80–0.86) for Banana 0.9

nnU-Net ≈ 0.90

DeepMedic ≈ 0.88–0.91

5× faster CPU-only inference

Hardware–efficiency trade-offs

### ****Reviewer #1 Comment 4 – Biomarker Module Uses Synthetic Data****

“Include plans to incorporate verified biomarker data.”

**Response:**
Added explicit future directions in **TriOx Module** description and **Future Work** (Lines 624–642):

Real-world biomarker integration

Validation against clinical tests

Cross-hospital EHR linkage

### ****Reviewer #1 Comment 5 – Statistical Openness****

“More detail on parameter distributions and Monte Carlo assumptions is needed.”

**Response:**
We added statistical detail in **Methods** (Lines 220–268), including:

Parameters and priors

Random seeds

Bootstrap confidence intervals

Prevalence assumptions

Stratified sampling

Full reproducibility path

### ****Reviewer #1 Comment 6 – Clinical Deployment Considerations****

“Enrich discussion of challenges in real-world environments.”

**Response:**
Added a new paragraph in **Discussion** (Lines 528–566) covering:

Staff training

Scanner variability

Artifact handling

Storage constraints

Connectivity challenges

### ****Reviewer #1 Comment 7 – Minor presentation and formatting issues****

**Response:**
All noted long sentences, spacing inconsistencies, and quotation formatting have been corrected.The manuscript underwent professional-level English polishing.

# ****Response to Reviewer #2****

Thank you for your thorough and insightful feedback. We have substantially strengthened the manuscript accordingly.

### ****Reviewer #2 Major Comment 1 – External/Clinical Validation****

“Manuscript lacks external validation beyond TCGA-STAD.”

**Response:**
A full **External Validation Study** using **AbdomenCT-1K TumorSubset (n=773)** has been added (Lines 342–389), including:

Cross-domain testing

Scanner/vendor diversity

CPU-only reproducibility

Log-file hashing

Zero-failure rate
This addresses generalizability and real-world heterogeneity.

### ****Reviewer #2 Major Comment 2 – Benchmarks with State-of-the-art Methods****

“Claims need benchmarking with standard metrics and uncertainty.”

**Response:**
We expanded **Comparison to Existing Tools** (Lines 398–429) to include:

Dice, CI

Sensitivity/specificity

Runtime

Compute cost

Interpretability trade-offs

This now situates Banana 0.9 within contemporary segmentation literature.

### ****Reviewer #2 Major Comment 3 – Statistical Rigor****

“Report variance, CI, sample-size justification, stratification, seeds, runs.”

**Response:**
Substantial updates in **Methods** (Lines 220–286):

Monte-Carlo assumptions clarified

CIs added for all metrics

Sampling justification

Error propagation

Reproducibility design

Exact seeds reported

All metrics now include SD or bootstrap CI.

### ****Reviewer #2 Major Comment 4 – Algorithmic Transparency (HU + Bayesian Updating)****

**Response:**
We added explicit algorithmic detail including:

Pseudocode-style descriptions (Lines 187–219)

Parameter ranges

Threshold calibration

YAML configs

Deterministic pipeline logic

This ensures full reproducibility.

### ****Reviewer #2 Major Comment 5 – Positioning Within Prior Work****

**Response:**
We significantly expanded the **Introduction** (Lines 71–142), covering:

Reproducible AI frameworks

Federated learning

Edge-AI medical imaging

Low-power inference literature

### ****Reviewer #2 Major Comment 6 – Ethics & Licensing****

**Response:**
Clarified IRB requirements and dataset licenses in **Ethics Statement** (Lines 547–553).
Clarified CC BY-NC 4.0 implications in **Data & Code Availability** (Lines 658–662).

### ****Reviewer #2 Major Comment 7 – Reproducibility Evidence****

**Response:**
We added:

Cross-platform reproducibility testing

Output-hash verification

CPU-only determinism

Public logs (773-case validation)

Documented in **Reproducibility** (Lines 429–456).

### ****Reviewer #2 Major Comment 8 – Expanded Limitations****

**Response:**
We added a more critical **Limitations** section (Lines 574–604), covering:

Synthetic biomarkers

Low-contrast CT

Rural hardware constraints

Domain shift

Dataset demographic limitations

### ****Reviewer #2 Minor Comments****

All addressed; includes:

Abstract restructuring

Improved keywords

Better figure/table captions

Clear pipeline diagram uploaded separately

Reduced repetition

Added missing references

# ****Final Statement****

We greatly appreciate the reviewers’ and editor’s time and detailed feedback.
We believe the revised manuscript is substantially strengthened, with:

**New external validation (773 cases)**

**Statistically rigorous design**

**Expanded benchmarking**

**Enhanced reproducibility documentation**

**Improved clinical applicability discussion**

**Clearer limitations and future roadmap**

We hope that the revised submission now meets the standards of PLOS ONE and respectfully submit the manuscript for further consideration.
