# Supplementary material for: Banana 0.9: An open-source, reproducible medical imaging system for low-resource gastric cancer screening
Source: PLoS One. 2026 Feb 24;21(2):e0339892. doi: 10.1371/journal.pone.0339892 (PMC12931783; doi:10.1371/journal.pone.0339892)
Supplement: Attachment — Submitted filename: The manuscript introduces Banana 0.docx [file pone.0339892.s004.docx]

The manuscript introduces Banana 0.9, an open-source, GPU-free CT imaging framework designed for low-resource gastric cancer screening. The work is clearly written, technically transparent, and well aligned with reproducibility principles. Public code, modular design, and full configuration files are strong strengths. However, a few points require clarification before acceptance:

1. **External validation:**

Since the AbdomenCT-1K subset lacks segmentation ground truth, the external experiment demonstrates reproducibility rather than accuracy. The authors should clarify this more explicitly and consider adding an indirect accuracy check or an additional dataset with annotations.

2. **Biomarker & clinical-risk modules:**

All risk parameters are literature-based rather than clinically measured. This should be more clearly framed as conceptual modeling, not validated clinical prediction.

3. **Cost-reduction claim:**

The ~60% cost savings need clearer assumptions (hardware pricing, lifespan, maintenance, electricity). A brief sensitivity analysis would strengthen this claim.

4. **HU-based segmentation limitations:**

The discussion should more directly acknowledge potential issues: low-contrast areas, scanner variability, and motion artifacts.

Overall, the manuscript is well presented and contributes meaningfully to open, reproducible medical imaging. With the above clarifications, it would be suitable for publication.
