# Supplementary material for: Banana 0.9: An open-source, reproducible medical imaging system for low-resource gastric cancer screening
Source: PLoS One. 2026 Feb 24;21(2):e0339892. doi: 10.1371/journal.pone.0339892 (PMC12931783; doi:10.1371/journal.pone.0339892)
Supplement: Attachment — Submitted filename: Response_to_Reviewers_PONE-D-25-49901_auresp_2.docx [file pone.0339892.s005.docx]

# ****Response to Reviewers****

Response to Reviewers — Revised Manuscript (R2 Final)

**Manuscript ID: PONE-D-25-49901R1**

**Title: Banana 0.9: An Open-Source, Reproducible Medical Imaging System for Low-Resource Gastric Cancer Screening**

Journal: PLOS ONE

**Reviewer #3 — Point-by-Point Response**

**Comment 1 — External validation clarity**

Reviewer comment:

“Since the AbdomenCT-1K subset lacks segmentation ground truth, the external experiment demonstrates reproducibility rather than accuracy. The authors should clarify this more explicitly and consider adding an indirect accuracy check or an additional dataset with annotations.”

Response:

We thank the reviewer for this important clarification. We fully agree that because AbdomenCT-1K does not contain voxel-level segmentation ground truth, the external experiment evaluates procedural reproducibility, not accuracy.

The manuscript has been revised accordingly:

Methods — External Dataset Validation

“Because the AbdomenCT-1K dataset does not include segmentation ground truth, this external validation evaluates procedural reproducibility and cross-platform determinism rather than absolute segmentation accuracy.”

Discussion — External Generalization

“As the dataset lacks voxel-level annotations, the external experiment is not an accuracy benchmark but a reproducibility stress test across heterogeneous scanners and acquisition protocols.”

Conclusions

“The external evaluation should be interpreted as a reproducibility assessment rather than a measure of segmentation accuracy, given the absence of ground-truth annotations in AbdomenCT-1K.”

These revisions ensure transparent interpretation of the external validation results.

**Comment 2 — Biomarker & clinical-risk modules**

Reviewer comment:

“All risk parameters are literature-based rather than clinically measured. This should be more clearly framed as conceptual modeling, not validated clinical prediction.”

Response:

We appreciate this suggestion and agree fully. We have added explicit statements describing the TriOx biomarker simulation and clinical risk-factor module as conceptual modeling tools, not validated predictors.

Revisions include:

Methods — Biomarker Module

“The TriOx biomarker and clinical-risk parameters represent a conceptual modeling framework derived from published literature, rather than a validated clinical prediction system.”

Limitations

“Because all risk parameters are literature-derived, the biomarker module should be interpreted as conceptual modeling rather than a clinically validated prognostic tool.”

**Comment 3 — Cost-reduction claim**

Reviewer comment:

“The ~60% cost savings need clearer assumptions (hardware pricing, lifespan, maintenance, electricity). A brief sensitivity analysis would strengthen this claim.”

Response:

Thank you for highlighting this point. We clarified the assumptions underlying the Monte Carlo cost estimations:

Results — Cost Simulation

“The estimated ~60% per-patient cost reduction assumes standard mid-range CPU hardware, a 3–5 year device lifespan, typical outpatient electricity costs, and no specialized GPU maintenance.”

Low-Resource Deployment

“The cost comparison reflects hardware-class assumptions (CPU vs. GPU acquisition cost, expected lifespan, and operating energy consumption) rather than absolute economic projections.”

This clarification ensures transparency regarding economic assumptions.

**Comment 4 — HU-based segmentation limitations**

Reviewer comment:

“The discussion should more directly acknowledge potential issues: low-contrast areas, scanner variability, and motion artifacts.”

Response:

We appreciate this comment. We expanded the manuscript to explicitly acknowledge these well-known limitations of deterministic HU-thresholding:

Comparison to Existing Tools

“Deterministic HU-based segmentation is sensitive to low-contrast lesions, motion artifacts, and scanner-specific noise patterns, which may reduce performance in certain clinical scenarios.”

Limitations

“HU-threshold segmentation may be affected by low-contrast boundaries, acquisition variability across institutions, and patient motion, underscoring the need for future hybrid HU + deep-learning approaches.”

**Reviewer #4**

Reviewer #4 stated:

“All comments have been addressed.”

No further action was required.

**General Manuscript Revisions**

To improve clarity and compliance with PLOS ONE guidelines, we also implemented the following updates:

**Structural Improvements**

Strengthened transitions between Results → Discussion → Conclusions.

Standardized section titles and terminology (e.g., “procedural reproducibility,” “deterministic HU segmentation”).

Language Polishing

Improved readability by shortening long sentences.

Ensured consistency in tense, voice, and formatting.

Technical Consistency

Verified internal consistency of numerical values, confidence intervals, dataset names, module descriptions, and table references.

Ensured Table 1 and Table 2 appear in the main manuscript as required.

Reproducibility Enhancements

Added clarifications regarding deterministic execution, hashing, fixed random seeds, runtime logs, and cross-platform reproducibility.

Confirmed that all code, configuration files, and documentation are publicly available under a CC BY-NC 4.0 license.

**Required Editorial Compliance Statements**

1. Data Availability Compliance

All data used in this study (TCGA-STAD, AbdomenCT-1K, and synthetic simulations) are publicly available, and no restricted-access data were used. Code, configuration files, and logs are openly accessible.

2. Figure Upload Policy

All figures have been removed from the manuscript body and are provided as separate high-resolution files as required by PLOS ONE.

3. Author Affiliation

The single author is now properly affiliated as Independent Researcher.

Recommended Citation Evaluation

Both reviewers provided methodological suggestions, but neither reviewer recommended any specific publications for citation.

We carefully reviewed all reviewer comments and confirmed that no additional references were explicitly requested.

Therefore:

No new citations were added in response to reviewer recommendations.

Existing citations remain appropriate and sufficient to contextualize the Banana 0.9 framework.

**Closing Statement**

We thank the reviewers and the academic editor for their constructive comments.

We believe the revisions have improved the clarity, transparency, and reproducibility of the manuscript, while maintaining its core contribution as an interpretable, GPU-free framework for low-resource medical imaging.

We respectfully submit this revised manuscript for further consideration by PLOS ONE.
